# Idiopathic intracranial hypertension exacerbated by aseptic meningitis from a nonsteroidal anti-inflammatory drug

**DOI:** 10.1016/j.ajoc.2026.102579

**Published:** 2026-04-07

**Authors:** Michael S. Trainer, Jonathan C. Horton

**Affiliations:** University of California, Department of Ophthalmology, San Francisco, USA

**Keywords:** Aseptic meningitis, Dural sinus stenosis, Ibuprofen, Idiopathic intracranial hypertension, NSAID, Papilledema, Partially empty sella

## Abstract

**Purpose:**

To report a case of hemorrhagic papilledema due to idiopathic intracranial hypertension, which was precipitated by aseptic meningitis from self-medication with a nonsteroidal anti-inflammatory drug (NSAID).

**Observations:**

A 29-year-old woman with obesity developed headache, emesis, and vision loss. Examination showed swollen optic discs surrounded by extensive hemorrhage. Neuroimaging revealed a partially empty sella, dilated optic nerve sheaths, and stenosis at the transverse-sigmoid sinus junction. A lumbar puncture yielded an elevated intracranial pressure (ICP), along with the unexpected finding of an aseptic meningitis. It was attributed to the patient's excessive use of ibuprofen to treat her headache.

**Conclusions and importance:**

Aseptic meningitis induced by an NSAID can precipitate an attack of idiopathic intracranial hypertension in a person at risk for this condition. Inflammatory cells in the meninges impair the absorption of cerebrospinal fluid (CSF), causing a rise in ICP that initiates the positive feedback cycle responsible for the pathogenesis of idiopathic intracranial hypertension.

## 1 Introduction

Meningitis is termed “aseptic” when bacterial or fungal cultures are negative. The most common etiology is viral infection, but it also can be caused by systemic disease, malignancy, autoimmune or inflammatory conditions, and medications. Drug-induced aseptic meningitis is associated with NSAIDs, antibiotics, intravenous immunoglobulins, and T3-receptor monoclonal antibodies^1^, ^2^, ^3^, ^4^. The cerebrospinal fluid (CSF) profile often mimics that of an infectious meningitis.^5^ Here we describe a patient with mild, undiagnosed idiopathic intracranial hypertension who came to medical attention when she developed aseptic meningitis induced by treating her cephalgia with high doses of ibuprofen.

## 2 Case presentation

A 29-year-old woman with a history of migraine, obesity (body mass index 37.44 kg/m^2^), and polycystic ovarian syndrome reported a one-month history of escalating headaches, vomiting, pulsatile tinnitus, transient visual obscurations, and blurred vision in her right eye. Her headache was worsened by recumbency. She experienced no relief from her usual triptan medication.

**Fig. 1:**
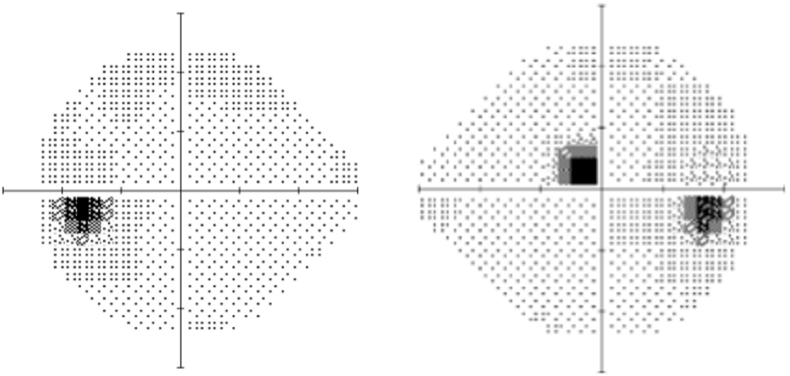
Humphrey visual field (24° except for 30° temporally) grayscale maps showing a deep parafoveal scotoma in the right eye.

**Fig. 2:**
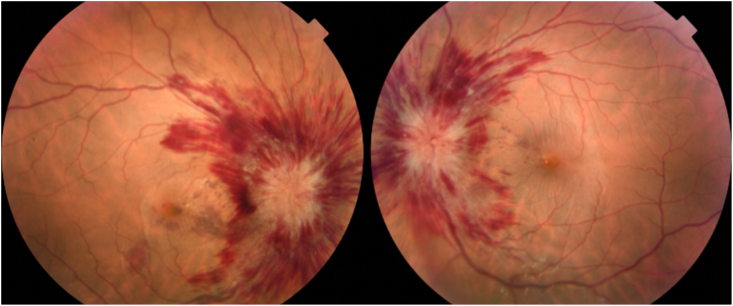
Fundus images showing papilledema with extensive hemorrhages radiating at least 15° along the retinal nerve fiber layer.

**Fig. 3:**
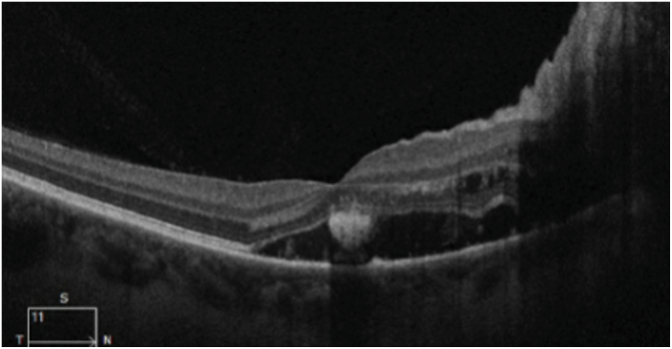
Ocular coherence tomography of the right eye showing subfoveal fluid, accounting for the reduction of visual acuity to 20/60.

She consulted an optometrist who noted a best-corrected visual acuity of 20/60 in the right eye and 20/20 in the left eye. Humprey visual fields showed a paracentral scotoma in the right eye (Fig. 1). The optic discs were swollen with massive peripapillary hemorrhage (Fig. 2). Optical coherence tomography (OCT) revealed macular edema in the right eye (Fig. 3), accounting for her reduced acuity.

**Fig. 4:**
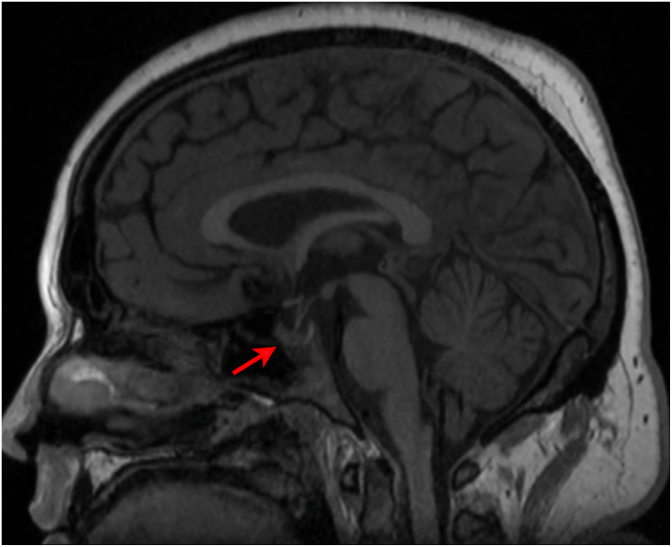
Sagittal T-1 weighted MR image showing a partially empty sella (arrow) at the time of clinical presentation, suggesting a history of chronic elevation of ICP.

**Fig. 5:**
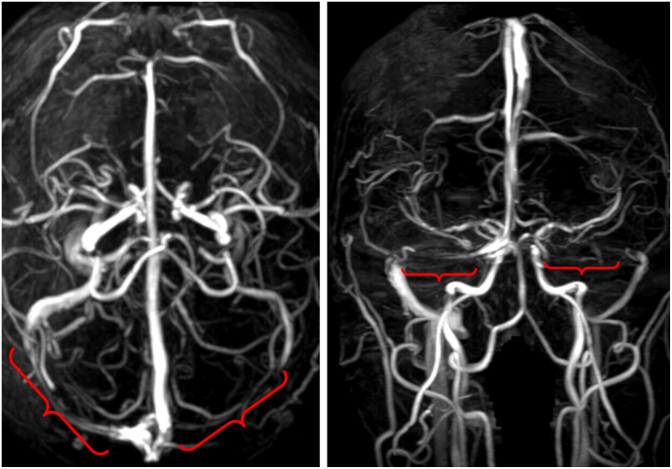
Axial (left) and coronal (right) MR venography showing a right dominant venous sinus system with severe bilateral stenosis of the transverse sinuses (red brackets).

The patient was referred immediately to our emergency room for further evaluation. The hematocrit, platelet count, prothrombin time, and international normalized ratio were normal. Brain magnetic resonance (MR) imaging with and without contrast was notable for bilateral optic nerve sheath dilation and a partially empty sella (Fig. 4). There was no intracranial mass lesion or abnormal dural enhancement. MR venography showed a right dominant transverse sinus, with marked bilateral stenosis (Fig. 5).

The history and findings pointed strongly to a diagnosis of idiopathic intracranial hypertension. For confirmation, a lumbar puncture was performed. It yielded an opening pressure of 33 cm H_2_O, 51 white blood cells (94% lymphocytes), 183 red blood cells, normal protein (24 mg/dL) and glucose (62 mg/dL). There were no oligoclonal bands and the IgG index was normal (0.5).

The unexpected discovery of a CSF pleocytosis led to a broad workup for an infectious cause. CSF bacterial, fungal, and acid-fast bacillus cultures were negative. Unbiased pathogen detection by metagenomic next-generation sequencing of the CSF was unremarkable. Serum rapid plasma reagin, Quantiferon Gold, and Lyme antibodies were negative.

On further questioning the patient revealed that she had been taking escalating doses of analgesics in the weeks preceding her diagnosis, eventually consuming more than 2000 mg of ibuprofen and 1000 mg of aspirin daily to control her cephalgia. After her workup revealed no other explanation, her CSF pleocytosis was attributed to chemical meningitis caused by excessive NSAID usage.

At the time of discharge she started treatment with 500 mg twice daily of extended release acetazolamide to manage her papilledema. A regimen of escalating doses of oral semaglutide was prescribed for weight loss. She was instructed to abstain from aspirin and NSAIDs, with a plan for a repeat lumbar puncture several months after discharge.

**Fig. 6:**
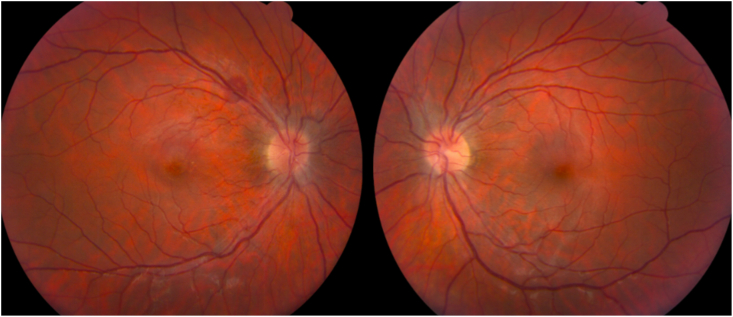
Fundus images showing nearly complete resolution of papilledema and hemorrhage 3 months later.

**Fig. 7:**
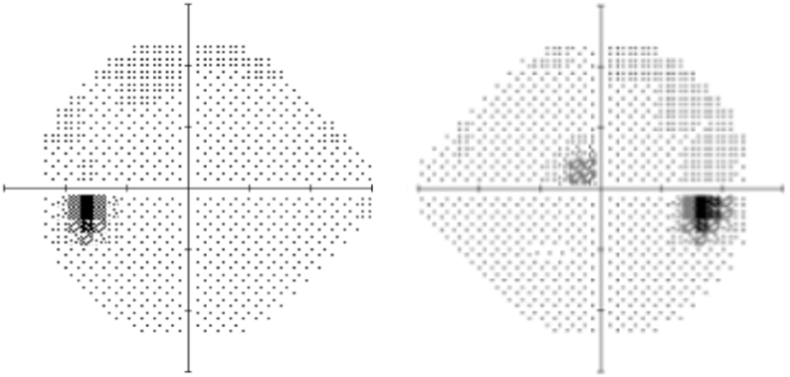
Humphrey visual field maps showing improvement in the parafoveal scotoma in the right eye 3 months later.

**Fig. 8:**
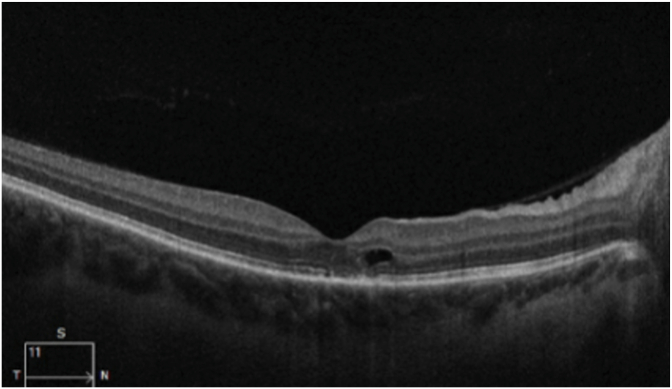
Optical coherence tomography of the right eye showing resorption of subfoveal fluid, explaining the improvement in acuity to 20/25.

Over the next three months the patient lost 30 pounds. Her visual acuity in the right eye improved to 20/25, with lessening of headache and tinnitus. Follow-up funduscopic examination (Fig. 6) showed nearly complete resolution of papilledema and peripapillary hemorrhage. The central scotoma in the right became more shallow (Fig. 7). OCT of the right macula showed a small residual focus of subretinal fluid inferonasal to the foveal pit (Fig. 8).

**Fig. 9:**
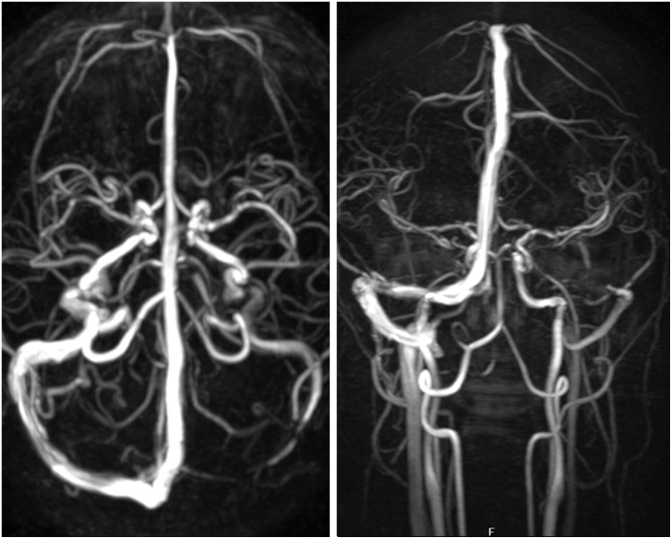
Axial (left) and coronal (right) MR venography showing resolution of right transverse sinus stenosis after ICP returned to normal.

The patient paused acetazolamide therapy for three days prior to a follow-up lumbar puncture, which yielded a normal opening pressure of 18 cm H_2_O, 3 white blood cells, 0 red blood cells, normal protein and glucose. Follow-up MR venography showed improvement in the right transverse sinus stenosis (Fig. 9).

## 3 Discussion

Papilledema is a term used exclusively to refer to swelling of the optic discs due to increased ICP.^6^ The most common cause in a general eye clinic is idiopathic intracranial hypertension.^7^ The workup should include MR imaging with and without contrast to exclude a space-occupying lesion. If negative, MR venography is useful to rule out venous sinus thrombosis or stenosis. The findings of dilated optic nerve sheaths, a partially empty sella, and stenosis at the transverse-sigmoid sinus junction are clues that ICP is elevated.^8^ A lumbar puncture is performed typically to measure the ICP and to ensure normal CSF constituents. Some authors have suggested that patients who fit the classic clinical profile of idiopathic intracranial hypertension, and have signs of raised ICP on MR imaging, may not require a lumbar puncture to confirm the diagnosis.^9^^,^^10^

Our patient did not conform to the classic clinical profile of idiopathic intracranial hypertension because of the appearance of her optic discs. Although peripapillary hemorrhage is commonplace in papilledema, the severity of the hemorrhage in our patient was wholly out of proportion to the degree of papilledema. Therefore, a lumbar puncture was mandatory. The extensive hemorrhages wreathing both optic discs suggested the possibility of a blood dyscrasia, Terson's syndrome, papillophlebitis, or an extreme spike in ICP that had occurred too recently for papilledema to develop fully. The lumbar puncture confirmed that ICP was elevated. It also led to the unexpected finding of a CSF pleocytosis. Although predominately lymphocytic, the latter was ultimately attributed to aseptic meningitis, and resolved after ibuprofen use was halted.^1^, ^2^, ^3^^,^^11^^,^^12^ The anticoagulant effects of ibuprofen combined with aspirin use were presumably responsible for the extensive peripapillary bleeding. It was a critical fundus observation, because it drove our decision to proceed with measurement of the opening pressure and analysis of the CSF.

Idiopathic intracranial hypertension is a relatively rare disorder, occurring in about 1/500 women of child-bearing age with obesity.^13^ Given the high prevalence of obesity, a large population is at risk for developing this condition. This fact raises an obvious question: what factors cause an obese but otherwise healthy person to develop idiopathic intracranial hypertension? The conversion from an asymptomatic state is thought to be driven by an auto-catalytic cycle.^14^^,^^15^ It begins with an elevation in jugular venous pressure caused by truncal obesity, which in turn raises pressure in the dural venous sinuses.^16^ As venous pressure rises, the absorption of CSF is reduced, elevating the ICP. This secondary rise in ICP acts to compress the dural venous sinuses, further increasing dural venous sinus hypertension. The narrow channel connecting the transverse sinus to the sigmoid sinus becomes a bottleneck, recognizable on MR imaging as a characteristic feature of idiopathic intracranial hypertension.

Bilateral transverse sinus stenosis is the most sensitive indicator of elevated intracranial pressure, found in 94% of patients with idiopathic intracranial hypertension and only 3-6.7% of healthy controls.^17^

In many patients, the positive feedback cycle triggering a rise in ICP is driven by a recent gain in weight.^18^^,^^19^ In our patient, we postulate that the instigating factor was chemical inflammation of the meninges caused by ibuprofen. Elevation in ICP, which occurs in only 14% of patients with aseptic meningitis, is strongly associated with an increased body mass index.^20^ This association suggests that overweight patients, who harbor subclinical findings from borderline intracranial hypertension, could be vulnerable to the development of papilledema when the meninges become inflamed. The partially empty sella imaged in our patient pointed to chronic ICP elevation. Her acute presentation was precipitated by worsening cephalgia, which she inadvertently exacerbated by self-medication with excessive doses of ibuprofen. The self-reinforcing cycle responsible for the emergence of intracranial hypertension was broken by stopping the drug, and treating with acetazolamide and semaglutide. Subsequently, the papilledema resolved, intracranial pressure returned to normal, and the caliber of the dural venous sinuses increased.^21^ NSAIDs are often used by patients to control headache caused by idiopathic intracranial hypertension. It is worth bearing in mind that rarely they can provoke an aseptic meningitis that makes the illness worse.

## CRediT authorship contribution statement

**Michael S. Trainer:** Writing – original draft, Investigation, Formal analysis, Data curation. **Jonathan C. Horton:** Writing – review & editing, Supervision, Funding acquisition, Formal analysis, Conceptualization.

## Patient consent

The patient consented in writing to publication of this case.

## Authorship

All authors attest that they meet the current ICMJE Criteria for Authorship.

## Funding

This work was supported by grants F30EY033201 (N.Y.T.), EY029703 (J.C.H.) and EY02162 (Vision Core Grant) from the National Eye Institute and by an unrestricted grant from Research to Prevent Blindness.

## Declaration of competing interest

The authors declare that they have no known competing financial interests or personal relationships that could have appeared to influence the work reported in this paper.
